# Strengthening governance and leadership of the national laboratory system in Liberia: achievements and challenges

**DOI:** 10.3389/fpubh.2025.1504451

**Published:** 2025-07-02

**Authors:** Torbandu H. Kohar, Ellen Munemo, Jonathan Kpaka, Nisha Marles, Ochiawunma Akwiwu-Ibe, Anicet G. Dahourou, Frantz Jean Louis

**Affiliations:** ^1^Liberia Ministry of Health, Monrovia, Liberia; ^2^Infectious Disease Detection and Surveillance Project of USAID, Monrovia, Liberia; ^3^National Public Health Institute of Liberia, Monrovia, Liberia; ^4^Infectious Disease Detection and Surveillance Project of USAID, Rockville, MD, United States

**Keywords:** laboratory governance, national laboratory policy, national laboratory strategic plan, diagnostic services, national laboratory systems

## Abstract

**Background:**

Liberia’s health system, severely impacted by prolonged conflict and the 2014–2015 Ebola outbreak, suffered from critical gaps in laboratory governance, workforce capacity, and diagnostic infrastructure. To address these challenges, the Ministry of Health (MoH), in collaboration with international partners, launched a national reform initiative aimed at strengthening the laboratory system. This study assesses the implementation and outcomes of the revised National Laboratory Policy and Strategic Plan introduced in 2019.

**Methods:**

A situational analysis was conducted to identify systemic weaknesses. The MoH formed a Technical Working Group to develop and implement the revised policy and plan. Qualitative and quantitative data were gathered through stakeholder interviews, document reviews, and laboratory quality audits.

**Interventions:**

The reform focused on five key areas: enhancing governance structures, expanding workforce development, implementing quality management systems (QMS), integrating private laboratories into the national network, and formalizing national laboratory policies and strategic guidelines.

**Results:**

Substantial improvements were observed across intervention areas. Governance was enhanced through strengthened roles of the National Diagnostic Division and National Public Health Institute of Liberia. A Bachelor of Medical Laboratory Science program was launched, with its first cohort enrolled in 2023. Eighteen laboratories joined a structured QMS program, and three attained a one-star rating. Four private laboratories were integrated into national disease surveillance. Comprehensive policy documents and infrastructure, equipment, and quality assurance guidelines were developed and disseminated.

**Conclusion:**

Liberia has made measurable progress in laboratory governance, workforce development, and quality assurance. Sustained investment in policy implementation, training, and infrastructure is critical to preserving these gains and improving national health security.

## Introduction

### Problem description

Over 14 years (1989 to 2003), Liberia endured two civil wars that left the health system fragmented and dysfunctional with the destruction of its infrastructure and a severely understaffed health workforce (1, 2). From 2014 to 2015, the country experienced the most devastating Ebola virus disease (EVD) outbreak in history. These tragic events stressed Liberia’s already fragile health system, resulting in a near collapse. The laboratory diagnostic network in Liberia was not excluded from the impact suffered across other parts of the health system. The laboratory network was not coordinated with national disease-specific control programs, and health facility-based laboratories were operating independently (3). The EVD outbreak highlighted laboratory services as a crucial priority for health security, emphasizing the urgent need for governance reform and system strengthening (3, 4). While these events underscored the need for systemic reform, they also highlighted the critical importance of laboratory governance as a cornerstone of health security.

### Available knowledge

Laboratory governance plays a key role in disease detection and response. Countries with weak laboratory policies face challenges in standardizing diagnostics, ensuring timely outbreak detection, and implementing quality assurance measures. In 2009, the Ministry of Health (MoH) established the National Diagnostic Division (NDD) with a mandate to provide leadership and governance to the laboratory network (5). The NDD was tasked with organizing, regulating, and overseeing all clinical laboratories in the country (5). These functions include policy development, rules and regulation enforcement, and accreditation support to provide quality laboratory services for the public (5, 6). The NDD worked in partnership with the National Public Health Reference Laboratory (NPHRL) and other institutions within the One Health framework to develop an integrated national diagnostic network capable of detecting, reporting, and responding to diseases. Liberia’s first National Laboratory Policy (2011–2015) and the National Laboratory Strategic Plan (2011–2014) aimed to standardize laboratory operations and establish a regulatory framework (7, 8). However, the 2014 EVD outbreak critically disrupted these efforts, rendering the NDD non-functional for over 2 years (2014–2016) (Supplementary Table 1). Shifting priorities, inadequate funding, and limited human resources prevented the full implementation of these policies, leaving the laboratory system without clear guidance (3).

### Rationale

A well-defined governance structure is essential for ensuring the strategic coordination of diagnostic services. The World Health Organization (WHO) defines governance as ‘a wide range of steering and rulemaking related functions carried out by governments as they seek to achieve national health policy objectives’ (9). Laboratory governance involves a systematic approach to building organizational capacity and ensuring sustainable, accessible, patient-centered, and quality-assured clinical testing services (10). Laboratory governance also includes clear lines of accountability for diagnostic services, standardized risk management policies, and professional guidelines for quality assurance (11). Effective laboratory governance requires national policies and strategic plans to centralize management, streamline operations, and enhance cost efficiency (12).

### Specific aims

Supported by the United States Agency for International Development’s (USAID) Infectious Disease Detection and Surveillance (IDDS) project, Liberia embarked on a journey to strengthen its laboratory diagnostic network. The initiative aligned with MoH objectives to expand leadership capacity, strengthen governance, and provide strategic guidance for laboratory services (1). Since 2019, IDDS has collaborated with the MoH to build laboratory capacity; develop infrastructure guidelines; and review laboratory policies, strategic plans, and standardization guidelines to strengthen the diagnostic network. This study aims to describe the efforts to rebuild and strengthen laboratory governance in Liberia through targeted capacity-building interventions and to evaluate the outcomes of these interventions on the laboratory system.

## Methods

### Context

The intervention was implemented by the MoH in collaboration with international partners such as USAID, WHO, and the Infectious Disease Detection and Surveillance (IDDS) project. The initiative aimed to align Liberia’s laboratory governance framework with global best practices while addressing country-specific challenges, including limited funding, workforce shortages, and infrastructure deficiencies.

### Interventions

#### Evaluation of past policies and strategic plans

- A review of the 2011 laboratory policy, 2011–2014 strategic plan, and reports from MoH and partners to assess initial objectives, successes, and challenges.
- Unstructured interviews with laboratory managers, technicians, and implementing partners to evaluate operational challenges and the impact of the EVD outbreak.

#### Establishment of the laboratory technical working group (TWG)

- A TWG formed by laboratory professionals, policymakers, and international donors was formed under the NDD leadership, bringing together key stakeholders from public and private institutions.
- From June to October 2019, the TWG conducted series of structured discussions to prioritize key areas needing urgent intervention.

#### Situational analysis of the laboratory landscape

- A comprehensive assessment of Liberia’s laboratory system was conducted by the Ministry of Health (MoH), supported by WHO, IDDS, and other development partners. This situational analysis aimed to identify the critical gaps in laboratory capacity, infrastructure, and service delivery. The assessment utilized standardized tools and frameworks, including WHO’s laboratory system strengthening guidance and Joint External Evaluation (JEE) diagnostic capacity indicators (13).
- The JEE assessment emphasized the need for a tiered laboratory system, enhanced coordination, and integration with surveillance systems, as well as clear governance structures to support sustainability. These findings informed the design and implementation of the revised National Laboratory Policy and Strategic Plan.

#### Development and adoption of standardized policies and guidelines

- TWG members collaboratively drafted and revised policy documents based on situational analysis findings.
- A validation workshop was conducted with regional health offices, private laboratories, and key stakeholders.
- The finalized National Laboratory Policy and Strategic Plan were officially adopted by the MoH on November 20, 2019, followed by nationwide dissemination.

### Study of the intervention(s)

An assessment was conducted to evaluate the impact of the interventions through stakeholder interviews, document reviews, and tracking of policy implementation.

### Measures

- Policies and strategic plans developed and adopted.
- Laboratory operational guidelines developed and adopted.
- Workforce capacity building program developed.
- Quality management system improved: Laboratory accreditation scores based on WHO-AFRO checklist.

### Analysis

A mixed-methods approach was used, combining qualitative data (stakeholder feedback, document reviews) with quantitative metrics (policy implementation rates, laboratory accreditation progress).

## Results

### Intervention implementation and evolution

The following sections outline the stepwise implementation of key interventions to strengthen laboratory governance in Liberia, guided by national policy and strategic objectives.

### The national laboratory policy and strategic plan were developed in 2019

A national laboratory policy defines the vision and mission of a country’s laboratory system and outlines roles and responsibilities for the planning and implementation of laboratory services (14). In addition, a laboratory strategic plan provides the corresponding roadmap, guides the practical implementation of the necessary laboratory system improvements, defines direction, sets standards, and prioritizes key system strengthening areas for resource allocation to achieve sustainable standards for quality laboratory services (15). The Liberia strategic documents set priority objectives for the laboratory system and facilitate the coordination of activities among the donors and the different implementing partners involved in laboratory projects at the national and sub-national levels. The documents are also used for planning and resource mobilization. The national laboratory policy clearly defined the roles and functions of the laboratory system components in Liberia. It included a legislative framework for national regulation, oversight, and management of the laboratory system. The national laboratory strategic plan set a roadmap for a sustainable national health laboratory system by streamlining the coordination and management of diagnostic services and defining mechanisms to strengthen laboratories’ preparedness for detection, surveillance, and response to epidemic-prone diseases listed in Liberia’s integrated disease surveillance and response guidelines (6, 16). In alignment with the strategic documents, specific institutional reforms were initiated to strengthen governance.

#### New governance structures were introduced to oversee laboratory operations

Laboratories require strong leadership and effective management to deliver accurate, timely and reliable test results. The national laboratory policy defined the roles and functions of the laboratory system components in Liberia. It included a national legislative framework for the regulation, oversight, and management of the laboratory system (5). The NDD was established as the technical arm of the MoH for laboratory services to oversee and support the clinical laboratory network and provide policy direction for the MoH (Supplementary Table 1). The National Public Health Institute of Liberia (NPHIL), established in January 2017, works to expand, and enhance the surveillance and response platforms and to strengthen public health diagnosis through the NPHRL (6). The NPHRL collaborates with the NDD to diagnose and monitor diseases of public health importance.

#### Public-private partnerships were established to integrate private laboratories into the national network

A public-private partnership (PPP) is often defined as a long-term contract between a private party and a government agency for providing a public asset or service (17). PPP is a cornerstone of health services delivery, including in the area of laboratory services strengthening in resources-limited countries (18). The success in developing and implementing the laboratory policy and strategic plan was stamped with a wide range of stakeholders’ footprints, from both public and private sectors, under the leadership of the NDD and the NPHRL. The situational analysis of the laboratory sector allowed the mapping of approximately 116 private laboratories across Liberia, providing different range of diagnostics tests (6). Public and private laboratories are included in Liberia’s strategic framework to build a resilient laboratory network system (5). The national laboratory policy provided the regulatory framework to streamline licensure processes and normalize private laboratories’ operations in Liberia (5). The private laboratory sector can play essential public health functions, such as participating in surveillance, workforce development, and disease control (9). In 2023, 4 private laboratories were included as sentinel sites for antimicrobial resistance surveillance (Supplementary Table 1).

### Standardized infrastructure and equipment guidelines were developed

Standardization and harmonization of testing menus, equipment, and consumables across the laboratory network can help improve the operation and efficiency of the laboratory system through rational prioritization of resources and more efficient supply chain management (12). The National Laboratory Standardization Guidelines document was developed in 2019. It detailed recommended testing menus, testing methods, equipment and consumables at each tier of the health system, and the optimal number and qualifications of staff required (19). In addition, to provide guidance on infrastructural development and improvement, the National Medical Laboratory Physical Infrastructure Guidelines was developed in 2020 (20) (Supplementary Table 1). These guidelines can be used by health administrators, laboratory managers, and supervisors when building or renovating laboratories to provide a safe working environment.

### Workforce capacity building program developed

Workforce capacity building through pre-service and in-service training programs is a core component of the establishment of a functional laboratory network system. Some of the challenges observed in Liberia were a shortage of qualified and skilled laboratory personnel, the absence of curricula for medical laboratory scientists and the lack of specialized training in the areas of leadership and management (3, 4). The national laboratory policy and the strategic plan established guidelines for pre-service and in-service training curricula and the continued professional development of laboratory personnel (5, 6). In 2020, IDDS collaborated with the University of Liberia College of Health Sciences to introduce the Bachelor of Medical Laboratory Science degree program and develop a national curriculum to standardize laboratory training across the country. The University of Liberia has started using the curriculum in training laboratory scientists. The first group was enrolled in 2023 (Supplementary Table 1). While qualitative improvements (e.g., new degree programs and national curricula) have been documented, quantitative outcome data such as graduate numbers or total staff trained are not yet available due to the recency of program implementation.

### Quality management system improved

To support the implementation of QMS, a quality manual template, which defines quality standards, management review, internal audits, identification and control of non-conformities and document control procedures was developed and can be customized by each laboratory. In 2021, IDDS worked with MoH, NDD, and NPHRL to publish the country’s first national integrated laboratory external quality assessment (EQA) plan. Liberia adopted the SLMTA based QMS program in 2017 and has mandated all laboratories to be enrolled to improve the quality of laboratory services in the country. Eighteen laboratories are enrolled in SLMTA, and local auditors are trained and are actively evaluating the progress of QMS implementation. Using the WHO-AFRO Stepwise Laboratory Improvement Process Towards Accreditation checklist, three county laboratories scored at least one star in 2022, and continued to work to improve the quality of their services (Supplementary Table 1).

### Formal adoption of laboratory policy and strategic plan documents

On November 20, 2019, the MoH officially received the strategic documents to help strengthen the Liberia’s laboratory system. The documents were handed over to the Minister of Health, by the USAID Mission. Following the handover, the NDD lead the dissemination of the documents. The NDD organized a series of workshops and meetings across the country to ensure that the new policies and strategic plans were communicated effectively to all relevant stakeholders. The NDD engaged with local health authorities and laboratory staff to explain the content and implications of the new documents. This engagement ensured that all parties understood their roles and responsibilities in implementing the updated policies and strategic plan.

## Discussion

### Governance structure

The implementation of Liberia’s National Laboratory Policy and Strategic Plan has significantly strengthened laboratory governance, improved quality assurance, expanded workforce capacity, and enhanced laboratory infrastructure. The introduction of structured policies and standardization measures has provided a regulatory framework that aligns with international best practices. The development of training programs, partnerships with private laboratories, and the implementation of external quality assessments have contributed to a more resilient laboratory system.

One of the major achievements of this initiative was the establishment of strong governance structures. The expansion of the NDD’s role and its collaboration with NPHIL have reinforced laboratory oversight, ensuring compliance with national regulations and improving coordination. The policy’s emphasis on public-private partnerships has further strengthened Liberia’s diagnostic service landscape, integrating private laboratories into the national network. This has improved access to diagnostic services, and promoted regulatory compliance.

### Workforce development

The lack of specialized training of laboratory professionals in leadership and management is one of the challenges faced by resource-limited countries (21, 22). The investment in workforce capacity development has also been a critical component of laboratory system strengthening. The introduction of a Bachelor of Medical Laboratory Science degree program at the University of Liberia College of Health Sciences has addressed the long-standing shortage of trained laboratory professionals. Additionally, the structured framework for continuous professional development and mentorship programs through the IDDS project have ensured ongoing skills enhancement, ultimately improving diagnostic service quality.

### Quality management systems (QMS)

The implementation of the WHO-AFRO SLIPTA framework and national EQA programs has led to measurable improvements in laboratory accreditation. The enrollment of 18 laboratories in the quality improvement initiative, with 3 achieving at least one-star accreditation status, demonstrates progress toward higher laboratory standards. The development of standardized infrastructure guidelines and equipment specifications has further streamlined laboratory operations and promoted best practices in quality management system.

### Challenges

#### Integration of private laboratories and financing strategies

Despite these advancements, several challenges remain. One of the most significant area for improvement identified in policies and plans is more attention to financing in developing laboratory improvement strategies (14). Financial sustainability is a major concern, as the laboratory system continues to rely on external funding for improvements. Private sector financing for health has historically been small and a coordinated strategy is needed to enhance PPP dialogs to leverage their financial contributions (23). Liberia has sought to leverage its sizable private health sector (approximately 45% of health facilities are privately operated) (23). Strengthening structured public-private collaboration by supporting and expanding the role of the Healthcare Federation of Liberia (HFL) as a platform for ongoing dialogue between government and private health stakeholders represent critical steps toward increasing domestic resource mobilization and financial protection in the health system (24). The Liberia Health Equity Fund (LHEF), was implemented to pool domestic revenues such as taxes and contributions into a sustainable mechanism for universal health coverage (25). The fund is intended to reduce out-of-pocket payments and ensure access to essential services. However, it remains at an early stage; by 2017, only 3% of Liberians had any form of health insurance (23).

#### Laboratory infrastructure and supply chain

Additionally, supply chain inefficiencies have affected the timely procurement of laboratory reagents and consumables, impacting service delivery. Variability in laboratory infrastructure across different regions necessitates continued investments to ensure equitable access to quality laboratory services. Efforts are underway to harmonize procurement with the National Essential Laboratory List and implement eLMIS tools (26).

### Limitations

This study is limited by its reliance on qualitative assessments and recent implementation timelines that restrict availability of longitudinal data. Specific clinical impact indicators, such as reduced turnaround times and expanded service coverage, were not systematically tracked and will require follow-up. Additionally, infrastructure disparities across counties were not mapped due to lack of disaggregated data. Looking ahead, building upon these gains requires strategic investments and sustained policy commitments.

### Future directions

To sustain progress, Liberia must accelerate the implementation of domestic health financing strategies. These include the expansion of PPPs to leverage private sector capacity, and integration of diagnostic services into national health insurance schemes. Building on the BMLS program’s early success, further investment in workforce development is essential to address chronic staffing shortages, strengthen QMS, and build in-country capacity to meet national and international accreditation standards. Moving forward, Liberia must prioritize long-term financial sustainability by increasing domestic funding for laboratory services. Strengthening supply chain mechanisms will be essential to reduce stockouts and enhance service continuity. Further efforts are also needed to expand the integration of laboratory services within the national health system, ensuring an efficient specimen referral system and a tiered network that meets the country’s diagnostic needs.

## Conclusion

Significant progress has been made in strengthening the leadership and governance of the national laboratory system in Liberia. The policy documents that are key in driving the laboratory network have been developed and disseminated and their implementation is ongoing. Ownership, leadership, and support within the MoH and NDD to sustain laboratory services as an integral part of their portfolio is a hallmark of the continuous achievements in laboratory capacity building in Liberia.

## Data Availability

The original contributions presented in the study are included in the article/Supplementary material, further inquiries can be directed to the corresponding author.

## References

[1] Ministry of Health Government of Liberia. Investment plan for building a resilient health system 2015 to 2021 (2015). Available online at: https://moh.gov.lr/wp-content/uploads/Liberia-Investment-Plan_-May-13_15-1.pdf (Accessed May 20, 2025).

[2] Mulbah E. Governance and health in Liberia. New York, NY: International Peace Institute (2016).

[3] Kennedy SB Wasunna CL Sahr P Bolay FK Dogba JB Eastman CB. The laboratory health system and its response to the Ebola virus disease outbreak in Liberia. Afr J Lab Med. (2016) 5:1–5. doi: 10.4102/ajlm.v5i3.509 PMC5433816 28879143

[4] Kennedy SB Dogba JB Wasunna CL Sahr P Eastman CB Mason GT. Pre-Ebola virus disease laboratory system and related challenges in Liberia. Afr J Lab Med. (2016) 5:1–5. doi: 10.4102/ajlm.v5i3.508 PMC5433815 28879142

[5] Ministry of Health and Social Welfare Republic of Liberia National Diagnostics Divison. National laboratory system policy of Liberia. Monrovia, Liberia: (2019).

[6] Ministry of Health and Social Welfare Republic of Liberia National Diagnostics Divison. Five-year strategic plan for the National Health Laboratory System of Liberia 2019–2024. Monrovia, Liberia: (2019).

[7] Ministry of Health and Social Welfare Government of Liberia National Diagnostics Unit. Strategic plan 2011-2014. Monrovia, Liberia: (2011).

[8] Ministry of Health and Social Welfare Government of Liberia National Diagnostics Unit. National laboratory policy for Liberia 2011-2015. Monrovia, Liberia: (2011).

[9] World Health Organization. (2015). Guidance for establishing a national health laboratory system. Brazzaville, ROC: World Health Organization. Regional Office for Africa.

[10] Freedman DB. Clinical governance—bridging management and clinical approaches to quality in the UK. Clin Chim Acta. (2002) 319:133–41. doi: 10.1016/S0009-8981(02)00034-7, PMID: 11955490

[11] Trenti T Canali C Scognamiglio A. Clinical governance and evidence-based laboratory medicine. Clin Chem Lab Med. (2006) 44:724–32. doi: 10.1515/CCLM.2006.130, PMID: 16729861

[12] Peter TF Shimada Y Freeman RR Ncube BN Khine AA Murtagh MM. The need for standardization in laboratory networks. Am J Clin Pathol. (2009) 131:867–74. doi: 10.1309/AJCPCBMOHM7SM3PJ, PMID: 19461096

[13] World Health Organization. Joint external evaluation of IHR core capacities of the Republic of Liberia: mission report, September 2016. Geneva: World Health Organization (2017).

[14] Ondoa P van der Broek A Jansen C de Bruijn H Schultsz C. National laboratory policies and plans in sub-Saharan African countries: gaps and opportunities. Afr. J. Lab. Med. (2017) 6:578. doi: 10.4102/ajlm.v6i1.578, PMID: 28879152 PMC5566126

[15] Nkengasong JN Mesele T Orloff S Kebede Y Fonjungo PN Timperi R. Critical role of developing national strategic plans as a guide to strengthen laboratory health systems in resource-poor settings. Am J Clin Pathol. (2009) 131:852–7. doi: 10.1309/AJCPC51BLOBBPAKC, PMID: 19461093

[16] Ministry of Health and Social Welfare Government of Liberia. Ministry of health Liberia national technical guidelines for integrated disease surveillance & response. Monrovia, Liberia: (2016).

[17] Yorfee LN (2019) The impact of corruption on governance and public-private partnership (PPP) in Liberia. Tilburg, Netherlands: Tilburg University.

[18] Alemnji G Zeh C Yao K Fonjungo P. Strengthening national health laboratories in sub-Saharan Africa: a decade of remarkable progress. Trop Med Int Health. (2014) 19:450–8. doi: 10.1111/tmi.12269, PMID: 24506521 PMC4826025

[19] Republic of Liberia Ministry of Health (2019) National laboratory standardization guidelines.

[20] Republic of Liberia Ministry of Health (2020) National medical laboratory physical infrastructure guidelines.

[21] Albetkova A Chaignat E Gasquet P Heilmann M Isadore J Jasir A. A competency framework for developing global laboratory leaders. Front Public Health. (2019) 7:199. doi: 10.3389/fpubh.2019.00199 31482080 PMC6710318

[22] Opio A Wafula W Amone J Kajumbula H Nkengasong JN. Country leadership and policy are critical factors for implementing laboratory accreditation in developing countries: a study on Uganda. Am J Clin Pathol. (2010) 134:381–7. doi: 10.1309/AJCP6KMOTCLISGJ3, PMID: 20716793

[23] Bryant Lee TF, Elise Lang achieving sustainable health financing in Liberia: prospects and advocacy opportunities for domestic resource mobilization the Global Fund, palladium; (2019). Available online at: https://thepalladiumgroup.com/downloads/154c298dc25924bda064f5b6cebdef46 (Accessed May 20, 2025).

[24] Africa Health Business. Unifying the private health sector in Liberia 2025. Available online at: https://africahb.com/unifying-the-private-health-sector-in-liberia/#:~:text=%28PPD%29%2C%20private,as%20well%20as%20business%20growth (Accessed May 20, 2025).

[25] Ministry of Health GoL (2016) Consolidated operational plan (FY 2016/17).

[26] Management science for heath. MSH and Liberian government launch new electronic logistics management information system. (2018). Available online at: https://msh.org/story/msh-and-liberian-government-launch-new-electronic-logistics-management/ (Accessed May 20, 2025).

