# Supplementary material for: Strengthening governance and leadership of the national laboratory system in Liberia: achievements and challenges
Source: Front Public Health. 2025 Jul 2;13:1504451. doi: 10.3389/fpubh.2025.1504451 (PMC12263591; doi:10.3389/fpubh.2025.1504451)
Supplement: Supplementary file 1 [file Data_Sheet_1.PDF]

Intervention Area	*Before Intervention*	*After Intervention*
Governance Structures	Fragmented oversight; no active national lab policy after 2015; NDD existed but was non-functional (2014–2016); Laboratories operated independently with no coordination.	Formal governance framework in place; National Lab Policy adopted in 2019 providing regulatory oversight. NDD revitalized as functional MoH arm overseeing labs in all 15 counties. Clear leadership and accountability established.
Workforce Development Initiatives	Severe shortage of qualified lab personnel; no standardized pre-service training for lab scientists (no bachelor-level training program); limited continuing education.	Workforce capacity expanded via new training frameworks. National curricula for pre-service and in-service training established. Continuous professional development programs and mentorship introduced. Notably, a BMLS degree program was launched to train skilled lab scientists (first cohort enrolled in 2023).
Bachelor of Medical Laboratory Science (BMLS) Program	**0** institutions offered a BMLS degree program in Liberia; only lower-level training available.	**1** institution (University of Liberia) running a national BMLS degree program (launched 2020) with a standardized curriculum. First student cohort enrolled in 2023, establishing a local pipeline for producing graduate-level laboratory scientists.
Quality Management Systems (QMS) Implementation	No structured QMS or accreditation program; labs not enrolled in any quality improvement scheme; zero labs accredited (0★ rating across the board).	SLMTA-based QMS program adopted in 2017; quality standards and EQA procedures implemented. 18 laboratories enrolled in the national QMS improvement program, 3 laboratories achieved at least a 1★ accreditation rating by 2022. Ongoing audits and mentorship in place to further improve quality.

Integration of Private Laboratories	Private labs (approximately 116 nationwide) unregulated and isolated from the national system; no formal integration or data sharing; **0** private laboratories involved in national surveillance.	Public-private partnership model established under new policy; licensing and oversight of private labs initiated. 4 private laboratories integrated as sentinel sites in the national antimicrobial resistance surveillance network by 2023.

Table 1: Summary of Laboratory System Strengthening Interventions – Before vs. After
